# Collateral Health Issues Derived from the Covid-19 Pandemic

**DOI:** 10.1186/s40798-020-00267-6

**Published:** 2020-08-08

**Authors:** Borja Muniz-Pardos, Jonathan Shurlock, Andre Debruyne, Juergen M. Steinacker, Mats Börjesson, Bernd Wolfarth, James L. J. Bilzon, Herbert Löllgen, Anca Ionescu, Petra Zupet, Michiko Dohi, Jeroen Swart, Victoriya Badtieva, Irina Zelenkova, Maurizio Casasco, Michael Geistlinger, Norbert Bachl, Fotini Tsofliou, Luigi Di Luigi, Xavier Bigard, Theodora Papadopoulou, Nick Webborn, Patrick Singleton, Mike Miller, Fabio Pigozzi, Yannis P. Pitsiladis

**Affiliations:** 1 GENUD (Growth, Exercise, Nutrition and Development) research group, University of Zaragoza, Zaragoza, Spain; 2 Brighton and Sussex University Hospitals, Brighton, UK; 3 European Federation of Sports Medicine Associations (EFSMA), Lausanne, Switzerland; 4 International Federation of Sports Medicine (FIMS), Lausanne, Switzerland; 5 Division of Sports and Rehabilitation Medicine, Ulm University Hospital, Ulm, Germany; 6 Department of Molecular and Clinical Medicine, Sahlgrenska Academy and Center for Health and Performance, Gothenburg University and Sahlgrenska University Hospital/Ostra, Gothenburg, Sweden; 7 Department of Sports Medicine, Humboldt University and Charité University School of Medicine, Berlin, Germany; 8 Department for Health, University of Bath, Bath, UK; 9 Sport Medical Center, Japan Institute of Sports Sciences, Tokyo, Japan; 10 UCT Research Unit for Exercise Science and Sports Medicine, Cape Town, South Africa; 11 I.M. Sechenov First Moscow State Medical University (Sechenov University), Ministry of Health of Russia, Moscow, Russian Federation; 12 Moscow Research and Practical Centre for Medical Rehabilitation, Restorative and Sports Medicine, Moscow Healthcare Department, Moscow, Russian Federation; 13 Italian Federation of Sports Medicine (FMSI), Rome, Italy; 14 Unit International Law, Department of Constitutional, International and European Law, University of Salzburg, Salzburg, Austria; 15 Institute of Sports Science, University of Vienna, Vienna, Austria; 16 Austrian Institute of Sports Medicine, Vienna, Austria; 17 Department of Rehabilitation, Nutrition and Sport Sciences, Bournemouth University, Bournemouth, UK; 18 Unit of Endocrinology, Department of Movement, Human and Health Sciences, University of Rome “Foro Italico”, Rome, Italy; 19 Union Cycliste Internationale (UCI), Aigle, Switzerland; 20 British Association Sport and Exercise Medicine, Doncaster, UK; 21 Defense Medical Rehabilitation Centre (DMRC), Loughborough, UK; 22 School of Sport and Service Management, University of Brighton, Eastbourne, UK; 23 World Olympians Association, Lausanne, Switzerland; 24 University of Rome “Foro Italico”, Rome, Italy; 25 Villa Stuart Sport Clinic, FIFA Medical Center of Excellence, Rome, Italy; 26 Collaborating Centre of Sports Medicine, University of Brighton, Eastbourne, UK

## Collateral Health Issues

At the end of 2019, a new coronavirus (Covid-19) outbreak occurred in Wuhan, China, and spread throughout the world despite efforts to contain the virus. At the end of January 2020, the General Director of the World Health Organization (WHO) declared a Public Health Emergency of International Concern, and by mid-May 2020, the worldwide number of known Covid-19 cases had surpassed 4.4 million including more than 300,000 deaths [1].

**Fig. 1:**
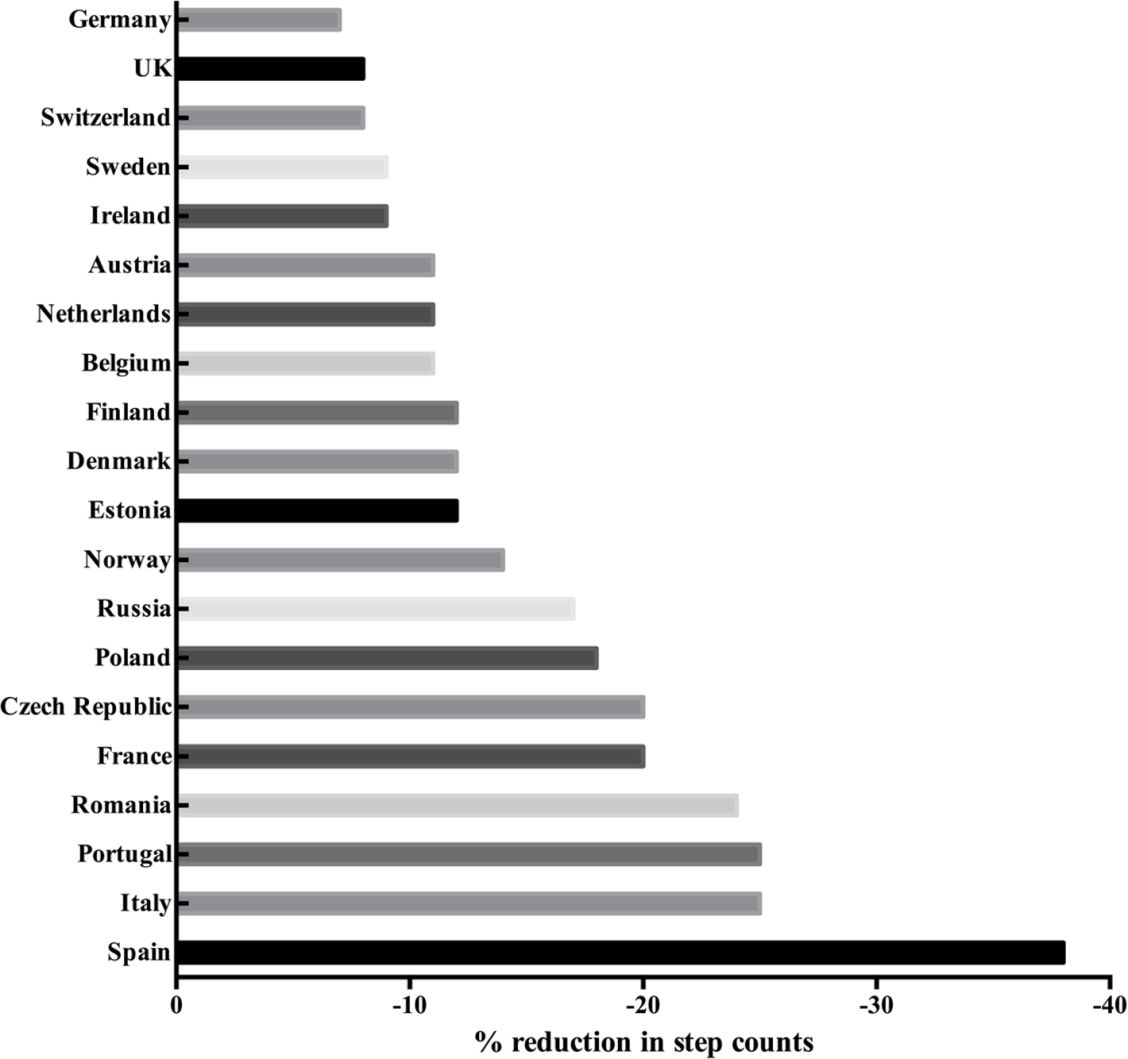
Reduction in physical activity levels in European countries dated the 22nd of March 2020. Data extracted from a report published by Fitbit Inc [4]

Currently, elderly individuals (i.e. > 65 years) and others suffering from respiratory diseases, diabetes, cancer, obesity, hypertension and cardiovascular diseases have been classified as populations at higher risk for disease severity and death due to Covid-19 infection [2]. Paradoxically, such individuals are also especially vulnerable to physical inactivity and lack of physical exercise. The enforced confinement renders it difficult for many individuals to adhere to the WHO recommendations on the minimal amount of physical activity (PA) (i.e. 150 min of moderate-intensity or 75 min of vigorous-intensity PA per week, or any equivalent combination of the two) [3]. A recent report by Fitbit Inc. demonstrates the severity of the Covid-19-related decline in PA in Europe as determined by the “number of steps” in over 30 million people, with a reduction in this proxy of PA ranging between 7 and 38% (Fig. 1) [4].

The impact of the Covid-19-related reduction in PA is worrying considering the already insufficient PA levels observed prior to the Covid-19 pandemic, with approximately 27.5% of adults not meeting the WHO recommendations for PA in 2016 [5]. This figure is however determined through self-reporting surveys and not more objective measurements (e.g. accelerometry), which is likely to bias and underestimate this prevalence. These numbers would be assuming that current WHO recommendations on the minimum weekly PA are sufficient for healthy adults and older adults, with some authors favouring more challenging minimum levels of 300 min per week as often as possible, or even more [6]. Notably, older individuals or medical patients unable to reach 300 min or even 150 min per week will aim to perform as much as their abilities and condition allow, in addition to balance and strength exercises [7]. Specific recommendations focused on decreasing sedentary behaviour (e.g. minimal amount of sedentary behaviour change required to produce meaningful health benefits [8]) are of crucial importance, especially during a lockdown period where the performance of moderate-vigorous PA can be challenging. A recent study has focused on the negative consequences of the lockdown on the health of the oldest age group (i.e. > 85 years and above) [9], which could further exacerbate sarcopenia, frailty, age-related functional decline and therefore an increased risk of chronic disease and associated all-cause mortality. This investigation highlighted the importance of increasing PA levels during lockdown through simple home-based exercises to reduce sedentary behaviour, to attenuate the decline in physical fitness and to optimize quality of life. The promotion of home-based activities that increase mobility such as gardening, mild housework and preparation of flavoursome meals can be used to break sedentary behaviour, increase levels of PA and muscular strength and enhance mood in older people [10], thus promoting health and wellbeing.

In addition to the negative impact of low PA levels or increased sedentary behaviour on individual health and fitness status, the fear of succumbing to Covid-19 seems to deter patients suffering from traditional medical issues (e.g. heart attacks, strokes and other medical emergencies) from seeking the treatment they may require, and this delay can compound further their health status, resulting in more heart attacks, strokes, and deaths [11]. A delay of only a few hours in a patient with an acute coronary syndrome seeking medical care can significantly impact patient survival [12], with a 30-min delay reducing average life expectancy by 1 year [13]. This compromised treatment strategy; whether due to a fear of infection or hospital saturation may threaten the life of many patients with no access to adequate treatment, intervention or rehabilitation, considerably increasing the number of deaths in the future.

The current need for social distancing compounds further the aforementioned issues. However, new policies that could include developing greater telemedicine capacity to rectify the lack of in-person treatment are urgently required. Such examples include hospital-at-home programmes that provide individuals requiring hospitalisation for reasons other than Covid-19 constant health care through a combination of virtual and in-person visits [14]. Such approaches could also be used to implement individualised exercise prescription or rehabilitation remotely in order to maintain physical fitness and improve health during the lockdown; physical exercise is the most economical remedy for a healthy society. This home-based exercise prescription supported by suitable technology could also target weight loss/prevention of weight gain as well as prevention of muscle loss in order to strengthen immune functions and reduce the severity of a potential infection, particularly considering recent evidence that obesity is an additional risk factor for Covid-19 [15].

Lockdown and social isolation have also been shown to have a profound impact on mental health [16]. Increases in alcohol consumption, drug abuse (especially anxiolytics and antidepressants), domestic and child abuse and online gambling during the lockdown reflect a high prevalence of mental health disruptions during this period. Additionally, the economic downturn and the associated increase in unemployment derived from the lockdown measures are likely to produce profound mental disorders and increases in suicide rates [16]. In sport, the inability of thousands of recreational and professional athletes to follow their physical training routines due to the enforced lockdown is triggering additional collateral health issues [17, 18]. The reduction in PA and exercise training during the lockdown limits the beneficial effects of exercise on depression, anxiety and mood, further compounding these adverse effects on mental health [19]. Mental health disorders are likely to occur or worsen especially in those elite athletes engaged in meticulous preparation for international events (e.g. Tokyo Olympics) as the postponement or cancellation of these events could ruin their last opportunity to compete at the top level.

While professional sport has been placed on hold, new technologies have played an important substitute role and several e-sports applications are being used worldwide to allow elite athletes and users at all levels to attenuate their fitness loss and also to socialise during the lockdown. For example, sports clubs have been very creative in offering virtual competitions via online platforms (e.g. Zwift, Strava or Bkool), which allow some athletes to train in simulated scenarios and maintain their fitness. As the lockdown restrictions are loosened in those countries which seem to be retarding the spread of the virus, professional sport under certain conditions is being reactivated, with boxing, football or basketball competitions among the first to be resumed in May–June 2020. Athletes from these contact and team sports are particularly vulnerable to the virus transmission due to the contact nature of the activity. The lockdown period has forced some international sports federations (e.g. FIFA) aiming to maintain the finishing dates of their leagues to concentrate a high number of competitions before the summer break, which translates into a saturated calendar of competitions implemented after several weeks of insufficient training. FIFA recently increased the maximum number of substitutions in a match from three to five hoping to prevent excessive injuries, although this mitigation may not be enough to safeguard the health of the athletes, especially after a long interruption of training.

Despite all these collateral issues derived from the Covid-19 lockdown, there are concurrently a number of positive factors impacting public health. A reduction in air pollution has been reported across 27 countries after 2 weeks of lockdown, and this improvement in air quality is estimated to have prevented 7400 premature deaths (all-cause) and 6600 cases of paediatric asthma [20]. Outdoor exercise, despite being recommended by different health organisations, has a number of adverse effects when performed in a polluted environment. High levels of pollutants can negatively impact on cardiorespiratory function, especially in those individuals suffering from cardiovascular or respiratory illnesses [21]. While current containing measures are unsustainable, such findings illustrate the great health benefits of reducing traffic-related air pollutant emissions, suggesting that periodic home-based activities/work (when/if possible) might be accompanied with rapid improvements of both air pollution and respiratory health, allowing for a safer opportunity to perform outdoor exercise.

The direct impact of the Covid-19 pandemic has been the main focus for health organisations in order to contain the spread of the virus, to set the most effective lockdown measures and to identify the best treatments or engineer a safe and effective vaccine. However, a focus on the collateral health- and sports-related issues is crucial as the health and economic consequences of not doing this may be even more catastrophic. There are many vital lessons to be learned beyond the rightly narrow focus to contain and manage the virus. There is an equally urgent need to manage all the collateral damage to public health caused by a worldwide pandemic in order to prevent the “cure” being worse than the disease.

## Data Availability

Not applicable.

## Abbreviations

PA: Physical activity
WHO: World Health Organization
